# High Fluorescent Porphyrin-PAMAM-Fluorene Dendrimers

**DOI:** 10.3390/molecules20058548

**Published:** 2015-05-13

**Authors:** Karla I. Garfias-Gonzalez, Ulises Organista-Mateos, Andrés Borja-Miranda, Virginia Gomez-Vidales, Simon Hernandez-Ortega, Sandra Cortez-Maya, Marcos Martínez-García

**Affiliations:** Instituto de Química, Universidad Nacional Autónoma de México, Ciudad Universitaria, Circuito Exterior, Coyoacán, México D.F. C.P. 04510, Mexico; E-Mails: karla_garfias@hotmail.com (K.I.G.-G.); ulises_orgma@outlook.com (U.O.-M.); borja.m.a@hotmail.com (A.B.-M.); gomvidal@unam.mx (V.G.-V.); shernandezortega@gmail.com (S.H.-O.); Sandra.crtz@gmail.com (S.C.-M.)

**Keywords:** dendrimers, fluorene, PAMAM, porphyrin

## Abstract

Two new classes of dendrimers bearing 8 and 32 fluorene donor groups have been synthesized. The first and second generations of these porphyrin-PAMAM-fluorene dendrimers were characterized by ^1^H-NMR, ^13^C-NMR, FTIR, UV-vis spectroscopy, elemental analyses and MALDI-TOF mass spectrometry. The UV-vis spectra showed that the individual properties of donor and acceptor moieties were preserved, indicating that the new dendrimers could be used as photosynthetic antennae. Furthermore, for fluorescent spectroscopy, these dendrimers showed good energy transfer.

## 1. Introduction

In the last few years, porphyrin-core dendrimer systems have been attracting much attention because the terminal substituent groups on the photoactive core can modulate their physico-chemical properties [1,2,3,4]. This has been extensively studied for a number of different applications, such as: shape-selective catalysts [3], organic solar cells [4], photoelectrochemical devices [5], light-emitting diodes [6], photodynamic therapy [7], chemical sensors [8], optical limiters [9], and so forth. In the past few years, there has been a considerable increase in the number of reports dealing with porphyrins decorated with dendrimers. A large number of different porphyrins bearing pendant linear oligofluorenyl arms have been reported [10,11,12] and it is possible to join them with PAMAM dendrimers. Poly(amidoamine) (PAMAM) species are a novel class of commercial dendrimers which possess an established molecular composition with different terminal functional groups. These macromolecules were first synthesized and investigated by Tomalia [1]. The design and modification of PAMAM dendrimers with fluorescent units could give them new interesting properties and expand their areas of application. In this paper we report on the synthesis and characterization of some new modified first and second generation PAMAM derivatives using diethylenetriamine-bearing fluorene fluorescent units at their peripheries and their photophysical characteristics.

## 2. Results and Discussion

**Scheme 1:**
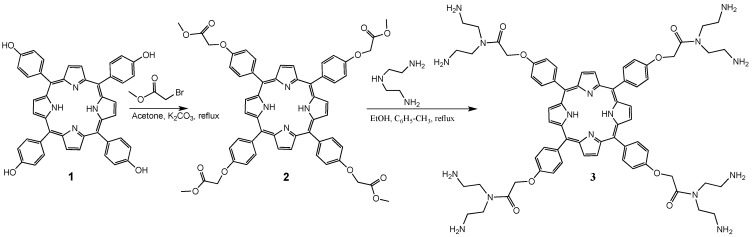
Synthesis of first-generation porphyrin dendrimer **3**.

Diethylenetriamine was used to obtain dendritic molecules with high molecular weight. Here, it is used as the building block in the synthesis of dendron-coated porphyrins. The porphyrin used was 5,10,15,20-tetrakis(4-hydroxyphenyl)-21*H*,23*H*-porphine, as a free base. The addition between the hydroxy groups of the porphyrin was conducted with methyl bromoacetate in acetone and K_2_CO_3_ (Scheme 1). Following with an amidation reaction with diethylenetriamine to obtain the first-generation dendrimer **3** (Scheme 1).

**Scheme 2:**
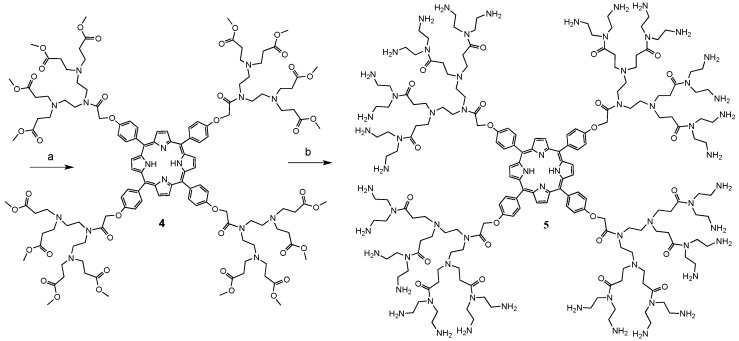
Synthesis of second-generation porphyrin-dendrimers: (**a**) methyl acrylate, methanol, reflux; (**b**) diethylenetriamine, methanol, toluene, reflux.

A mixture of the amide **3** and methyl acrylate in methanol was stirred in a nitrogen atmosphere at 90 °C. The MeOH and excess of methyl acrylate, as volatiles, were evaporated on a rotary evaporator, thus obtaining compound **4**. Finally the second-generation dendrimer **5** was obtained from compound **4** with an excess of diethylenetriamine in a mixture of methanol and toluene 1:1 at reflux for 24 h. (Scheme 2). It should be pointed out that compounds **2**, **3**, **4** and **5** have good solubility in most organic solvents.

**Scheme 3:**
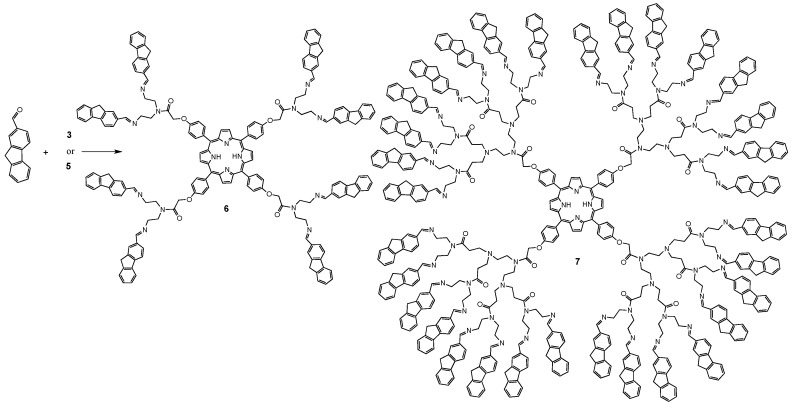
Synthesis of dendrimers **6** and **7** with peripheral fluorene moieties.

Finally the fluorene-2-carboxaldehyde moiety was attached to the porphyrin-PAMAM dendrimers in methanol/benzene 1:1 at reflux for 3 days. The compounds **6** and **7** were washed with methanol to remove the excess of fluorene (Scheme 3).

**Figure 1:**
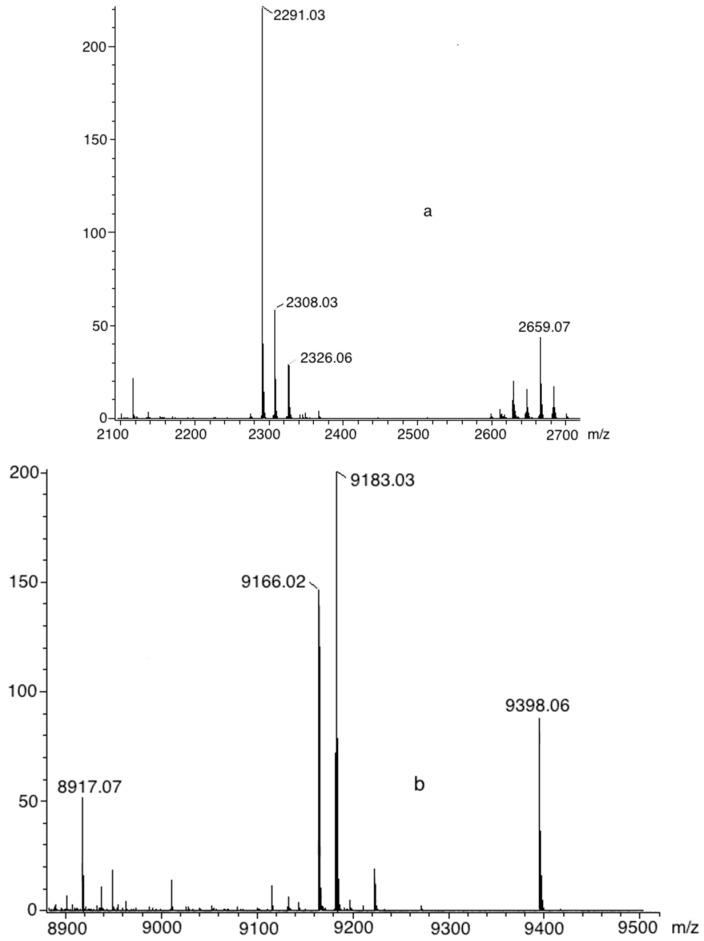
MALDI-TOF mass spectra of dendrimers **6** (**a**) and **7** (**b**).

Complete functionalization of the porphyrin was confirmed using NMR (^1^H, ^13^C) and matrix assisted laser desorption ionization time-of-flight mass spectrometry (MALDI-TOF-MS). In Figure 1, the MALDI-TOF mass spectra for dendrimers **6** and **7** show a peak at *m/z* = 2659 and 9398, respectively.

### 2.1. Optical Properties in Solution

**Figure 2:**
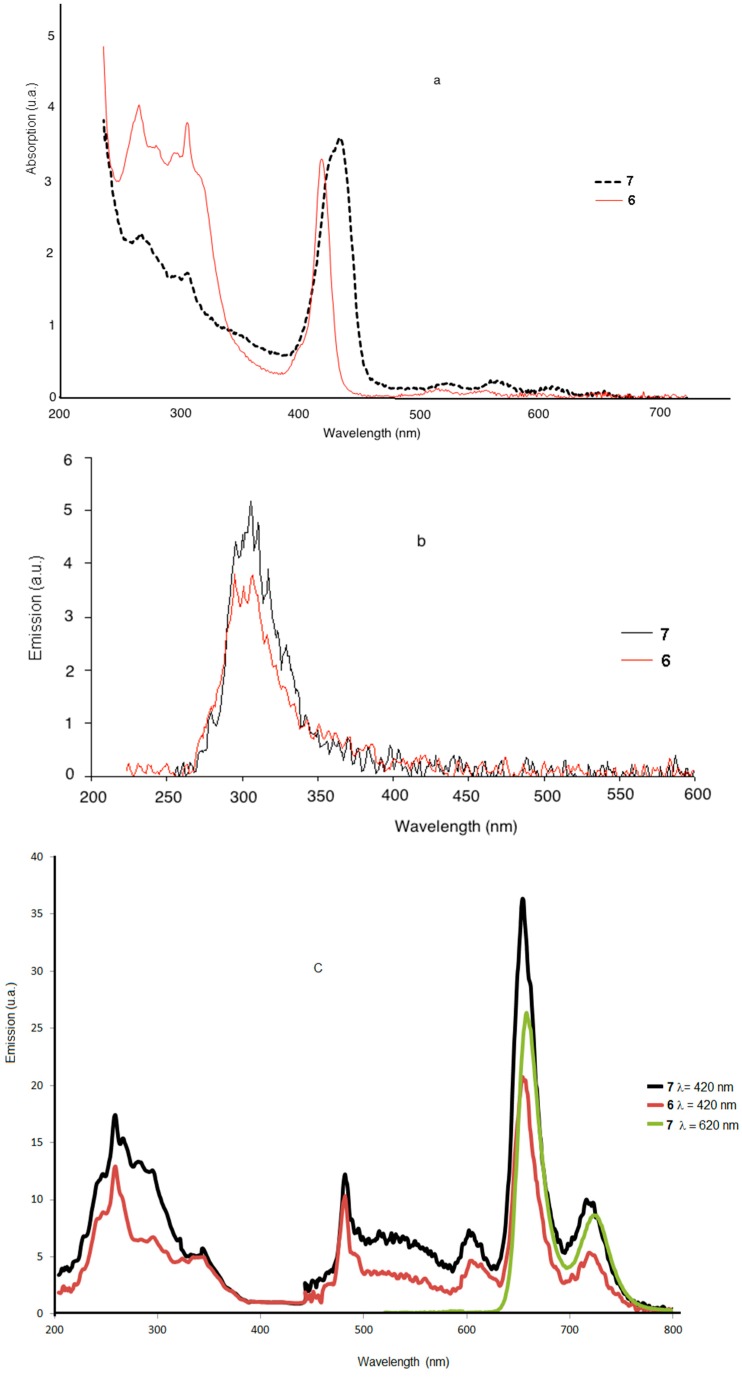
(**a**) Absorption (**b**) and (**c**) Fluorescence in chloroform at room temperature (λ_ex_ 250, 420 and 620 nm) of **6** and **7** in CH_2_Cl_2_ at room temperature.

The absorption spectra of **6** or **7** contain the characteristic fluorene group peaks 272 and 309 nm, as well as the porphyrin Soret band at 419 and 436 nm and the Q bands between 557 and 655 nm for the first and second generation dendrimers, respectively (Figure 2a). The spectra are quite similar, but the maximum absorption spectrum of the second-generation dendrimer is higher and broader than the spectrum of the first generation dendrimer. This band is also slightly red shifted compared to 419 nm for the first generation, but not as much as for the second generation (436 nm). This could be due to presence of the 32 fluorene moieties and the length of the PAMAM spacer groups.

The dendrimers **6** and **7**, after excitation at 250 nm, showed one broad band at 300 nm, the intensity of which was higher for the second-generation dendrimer **7** than for the first generation dendrimer **6** (Figure 2b). When the dendrimers are excited at 420 nm in the Soret band, the emission spectra reveal a strong red fluorescence with a peak maximum at 653 nm and a weak shoulder at 716 nm (Figure 2c) and almost no residual emission of the fluorene for both compounds. Thus, there is apparently a good energy transfer between fluorene and porphyrin, since the essentially red emission of the porphyrin is observed in comparison to blue emission of fluorene at around 300 nm. 

In fact, the porphyrin core emits almost all the UV energy absorbed by the fluorene units. The excitation spectra obtained after exciting in the strongest emission band at around 620 nm reveal an emission from the Soret state, the three first Q states, as well as from the fluorene. This indicates that excitation over all the 250–620 nm region leads to all the fluorescent excited states of the porphyrin. Table 1 shows the absorption spectral data of compounds **6** and **7** in CH_2_Cl_2_ at 24 °C, where the molar extinction coefficient (ε) of the absorbance of the dendrimers **6** and **7** showed an increase in the second-generation dendrimer **7**, while the absorption maximum (λ_max_) for the dendrimers **6** and **7** was slightly red shifted from 419 to 436 nm (solvatochromism) with an increase in ε.

**Table 1:** Summarized absorbance data (CH_2_Cl_2_).

Sample	Fluorene_max_ (nm)	Soret Band_max_ (nm)	Q_max_ (nm)	ɛ (×10^−5^ M^−1^ cm^−1^)
**6**	272, 309	419	557	0.235
**7**	266, 307	436	564	0.247

### 2.2. EPR Studies

**Figure 3:**
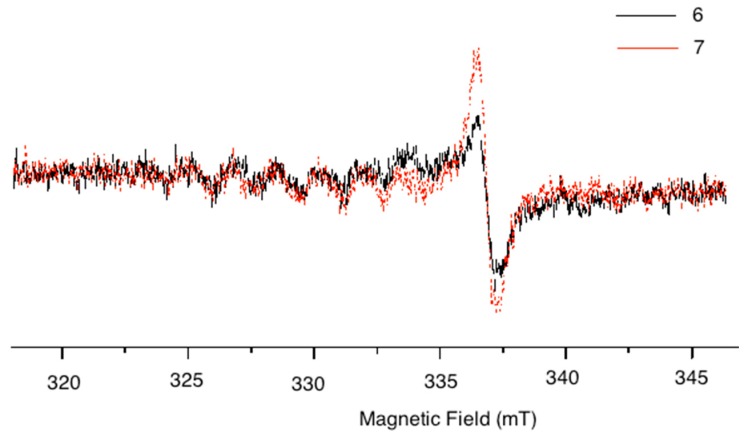
EPR spectra of compounds **6** and **7**.

Porphyrins have an extensive system of delocalized π electrons. The EPR signals of porphyrin-compounds are generated by the interaction of the delocalized π electrons of the porphyrin with the magnetic field. The EPR spectra of fluorene-porphyrins **6** and **7** are shown in Figure 3.

In the EPR spectrum of the solid sample, the overlap of two signals in the central field was observed. One of them corresponds to a singlet with g = 2.0036; this signal has been assigned to the formation of oxygen radicals by the interaction of O_2_ with the porphyrin ring [13], but the signal intensity is sensitive to the saturation effect of the microwave power related to the isoelectric point (pH value) [14].

The other signal presents hyperfine structure due the interaction of the unpaired electron with the magnetic cores (mainly nitrogen atoms) of the porphyrin macrocycle, (nitrogen atoms I_N_ = 1 and hydrogen atoms I_H_ = 1/2) [14], this signal exhibited nine lines due to the coupling between the porphyrins’ nitrogen atoms due to coupling of unpaired electrons with the conjugated porphyrin system [15]. The low intensity of this signal agrees with the presence of an electron-withdrawing group linked to the porphyrin macrocycle ring [16].

### 2.3. Crystal Structure Determination

**Figure 4:**
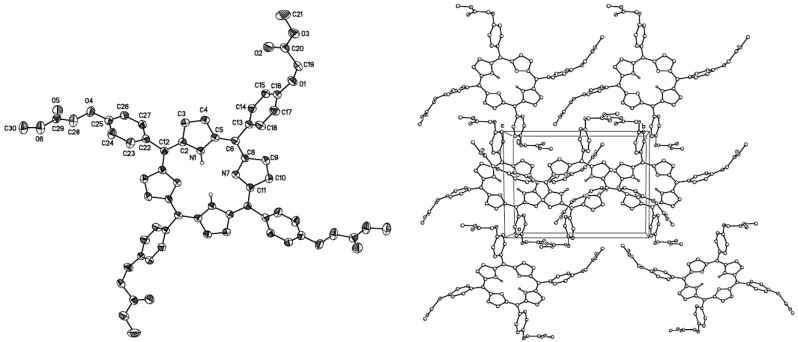
Crystal structure and crystal-packing of dendrimer **2**.

The structure of dendrimer generation 0.5 molecule **2** was confirmed by X-ray diffraction analysis of a single crystal prepared by recrystallization from chloroform at room temperature. The perspective view and crystal-packing diagram of molecule **2** are shown in Figure 4.

The suitable crystal of compound **2** was rolled in epoxy resin and mounted on glass fiber. A Bruker Apex AXS CCD area detector X-ray diffractometer was the instrument used for the determination. The data were first reduced and corrected for absorption using psi-scans and then solved using the program SHELL-XS. All non-hydrogen atoms were refined with anisotropic thermal parameters and the hydrogen atoms were refined at calculated positions with thermal parameters constrained to the carbon atom on which they were attached. A summary of the key crystallographic information is given in Table 2. 

**Table 2:** Crystal data and structure refinement.

Empirical formula	C_56_ H_46_ N_4_O_12_
Formula weight	966.97
Temperature	298(2) K
Wavelength	1.54178 Å
Crystal system	Monoclinic
Space group	P 2_1_/c
Unit cell dimensions	a = 12.3084(3) Å α = 90°
	b = 16.4548(4) Å β = 98.2840(10)°
	c = 11.8932(2) Å γ = 90°
Volume	2383.62(9) Å^3^
Z	2
Density (calculated)	1.347 Mg/m^3^
Absorption coefficient	0.789 mm^−1^
F(000)	1012
Crystal size	0.364 × 0.105 × 0.017 mm^3^
Theta range for data collection	3.629 to 68.264°
Index ranges	−14 ≤ h ≤ 14, −19 ≤ k ≤ 19, −14 ≤ l ≤ 12
Reflections collected	27,160
Independent reflections	4359 [R(int) = 0.1102]
Completeness to theta = 67.679°	99.8%
Refinement method	Full-matrix least-squares on F2
Data/restraints/parameters	4359/1/330
Goodness-of-fit on F2	1.048
Final R indices [I > 2sigma(I)]	R1 = 0.0668, wR2 = 0.1691
R indices (all data)	R1 = 0.0988, wR2 = 0.1936
Largest diff. peak and hole	0.747 and −0.269 e·Å^−3^

CCDC 1063702 contains the supplementary crystallographic data for this paper. These data can be obtained free of charge via www.ccdc.cam.ac.uk/conts/retrieving.html (or from the CCDC, 12 Union Road, Cambridge CB2 1EZ, UK; fax: +44-122-333-6033; E-Mail: deposit@ccdc.cam.ac.uk.

## 3. Experimental Section

### 3.1. General Information

Solvents and reagents were purchased as reagent grade and used without further purification. Ether and toluene were distilled from sodium and benzophenone, acetone was distilled over calcium chloride, and methanol was distilled over calcium oxide powder. Column chromatography was performed on Merck silica gel 60 Å (70–230 mesh). ^1^H- and ^13^C-NMR spectra were recorded on a Varian Unity-300 MHz instrument with tetramethylsilane (TMS) as an internal reference. Infrared (IR) spectra were measured on a Nicolet FT-SSX spectrophotometer. Elemental analysis was determined by Galbraith Laboratories, Inc. (Knoxville, TN, USA). Matrix-assisted laser desorption ionization time-of-flight mass spectroscopy (MALDI-TOF-MS) was performed on a JEOL JMS AX505 HA instrument using 9-nitroanthracene (9NA) as a matrix. The UV-Vis absorption spectra were obtained at room temperature with a Shimadzu 2401 PC spectrophotometer, while a Perkin-Elmer LS-50 spectro-fluorimeter was used for the fluorescence spectra. The excitation wavelengths used for the emission properties are reported in the text. The EPR measurements were made in a quartz tube at room temperature in the solid sample, with a Jeol JES-TE300 spectrometer operating in X-Band fashion at 100 KHz modulation frequency and a cylindrical cavity in the mode TE_011_. The external calibration of the magnetic field was made with a Jeol ES-FC5 precision gauss meter and microwave frequency with a HP 5350B frequency counter. Spectral acquisition, manipulations and simulation were performed using the program ES-IPRITS-TE. The EPR spectrum was recorded as a first derivation.

### 3.2. Synthesis of Generation 0.5 Dendrimer **2**

A mixture of the methyl bromoacetate (1.60 mmol), potassium carbonate (3.20 mmol mmol) in dry acetone (50 mL) was heated to reflux and stirred vigorously in a nitrogen atmosphere for 20 min. Compound **1** (1.60 mmol) dissolved in dry acetone (40 mL) was added dropwise and the reaction was continued for 6 hours. The mixture was filtered. The filtrate was evaporated to dryness under pressure. The residue dissolved in diethyl ether was washed with an aqueous solution of 5% Na_2_CO_3_ (three times). The organic layer was dried and evaporated to give the ester **2** (0.27 mmol) as a purple solid in 90% yield. m.p: ˃300 °C. FTIR (pellet, KBr, cm^−1^): 2954, 2920, 1750, 1602, 1503, 1430, 1298, 1207, 1173, 1140, 1076, 963, 806, 708, 604, 593. UV-vis (CH_2_Cl_2_): λ_max_ 418, 447, 515, 548, 591, 650. ^1^H-NMR (CDCl_3_) δ_H_: −2.77 (br, 2H, NH), 3.95 (s, 6H, O-CH_3_), 4.96 (s, 8H CH_2_-O), 7.30 (d, 8H, Ar, *J* = 8.7 Hz), 8.16 (d, 8H, Ar, *J* = 8.7 Hz), 8.87 (br, 8H, pyrrole). ^13^C-NMR (CDCl_3_) δ_C_: 29.6 (C-CO), 32.4 (O-CH_3_), 68.0 (CH_2_-O), 112.9 (Ar), 119.4 (Ar_ipso_), 130.9 (pyrrole), 135.6 (Ar), 135.7 (pyrrole_ipso_), 157.63(Ar_ipso_-O), 169.4 (C=O). MS (FAB^+^) *m/z*: 966 (M^+^). Anal. Calcd. for C_56_H_46_N_4_O_12_. C 69.56%, H 4.79%, N 5.79% O 19.85%. Found: C, 69.56; H, 4.77%.

### 3.3. Synthesis of Generation 1.0 Dendrimer **3**

A mixture of ester-containing derivative **2** (0.22 mmol) and diethylenetriamine (0.92 mmol) in methanol (15 mL) and toluene (15 mL) were refluxed for 24 h in an atmosphere of nitrogen. The organic solvent and the excess diethylenetriamine were removed *in vacuo.* The residue was washed several times with alcohol to obtain the amide compound **3** (0.16 mmol) as a purple solid in 85% yield. m.p: ˃300 °C. FTIR (pellet, KBr, cm^−1^): 3297, 2938, 1651, 1545, 1470, 1348, 1290, 1235, 1176, 1120, 797, 731, 587. UV-vis (CH_2_Cl_2_): λ_max_ 206, 426, 518, 559, 603, 647. ^1^H-NMR (CDCl_3_) δ_H_: −2.79 (br, 2H, NH), 1.90–1.96 (m, 16H, CH_2_-NH_2_), 3.30–3.54 (m, 16H, CH_2_-NH_2_), 4.90 (s, 8H, CH_2_-O), 7.41 (br, 8H, Ar), 8.08–8.11 (br, 8H, Ar), 8.85 (br, 8H, pyrrole). ^13^C-NMR (CDCl_3_) δ_C_: 38.8 (CH_2_-NH_2_), 40.4 (CH_2_-NH_2_), 41.6 (CH_2_-NH_2_), 43.4 (CH_2_-NH_2_), 53.7 (CH_2_-N), 57.8 (CH_2_-N), 58.4 (CH_2_-O), 69.1 (CH_2_-O) 108.6 (Ar), 115.0 (Ar_ipso_), 137.3 (pyrrole), 164.6 (C=O), 171.9 (C=O), 175.2 (C=O), 175.6 (C=O). MS (FAB^+^) *m/z*: 1,250 (M^+^). Anal. Calcd. for C_68_H_82_N_16_O_8_. C 65.26%, H 6.60%, N 17.91%. Found: C, 65.6; H, 6.58%.

### 3.4. Synthesis of Generation 1.5 Dendrimer **4**

A mixture of the amide **3** (0.14 mmol) and methyl acrylate (2.32 mmol) in methanol (20 mL) was stirred in a nitrogen atmosphere at 90 °C for 48 h. Methanol and excess methyl acrylate were removed *in* *vacuo*. The residue was washed with hexane and methyl acetate to obtain the ester compound **4** (0.12 mmol) as a purple oil in 80% yield. FTIR (film, cm^−1^): 3391, 3320, 2952, 2847, 1732, 1672, 1606, 1507, 1353, 1243, 1177, 1053, 992, 966, 844, 805, 735, 605. UV-vis (CH_2_Cl_2_): λ_max_ 421, 454, 518, 554, 594, 649, 685. ^1^H-NMR (CDCl_3_) δ_H_: −2.74 (br, 2H, NH), 2.53 (m, 32H, CH_2_-CO), 2.83 (m, 32H, CH_2_-N), 2.60 (m, 16H, CH_2_-N), 3.69 (m, 16H, CH_2_-NCO), 3.72 (m, 48H, O-CH_3_), 4.86 (s, 8H CH_2_-O), 7.41 (d, 8H, Ar, *J* = 8.7 Hz), 8.20 (d, 8H, Ar, *J* = 8.1 Hz), 8.90 (br, 8H, pyrrole). ^13^C-NMR (CDCl_3_) δ_C_: 29.67 (CH_2_-CO), 32.47 (CH_2_-NCO), 36.81 (CH_2_-CO), 49.69 (CH_2_-N), 51.59 (CH_3_-O), 53.2 (CH_2_-N), 67.62 (CH_2_-O), 113.1 (Ar), 119.4 (Ar_ipso_), 131.1 (pyrrole), 135.7 (Ar), 157.25 (Ar_ipso_), 168.1 (C=O), 172.9 (C=O), 173.4 (C=O). MS MALDI TOF *m/z*: 2627 (M^+^). Anal. Calcd. for C_132_H_178_N_16_O_40_. C 60.31%, H 6.82%, N 8.52%. Found: C, 60.31; H, 6.82, N, 8.54%.

### 3.5. Synthesis of Generation 2.0 Dendrimer **5**

A mixture of ester-containing derivative **4** (0.11 mmol) and diethylenetriamine (2.31 mmol) in methanol (15 mL) and toluene (15 mL) were refluxed for 24 h in an atmosphere of nitrogen. The organic solvent and the excess diethylenetriamine were removed *in vacuo.* The residue was washed several times with alcohol to obtain the amide compound **5** (0.09 mmol) as purple oil in 82% yield. (film, cm^−1^): 3257, 3074, 1644, 1547, 1433, 1368, 1293, 1239, 1178, 1043, 998, 642, 597. UV-vis (CH_2_Cl_2_): λ_max_ 209, 418, 517, 555, 593, 648. ^1^H-NMR (CDCl_3_) δ_H_: −2.74 (br, 2H, NH), 1.92–2.00 (m, 64H, CH_2_-NH_2_), 2.73–2.78 (m, 16H, CH_2_-N), 2.86–2.90 (m, 16H, CH_2_-N), 2.98 (m, 64H, CH_2_-N), 3.05–3.18 (m, 16H, CH_2_-N), 3.61 (s, 32H, CH_2_-N), 3.95 (s, 8H, CH_2_-NCO), 3.99 (s, 8H CH_2_-NCO), 4.86 (s, 8H CH_2_-O), 7.15–7.47 (m, 8H, Ar) 8.05–8.16 (m, 8H, Ar), 8.52 (br, 2H, pyrrole), 8.65 (br, 2H, pyrrole), 8.85 (br, 4H, pyrrole). ^13^C-NMR (CDCl_3_) δ_C_: 21.2 (CH_2_-NH_2_), 38.9 (CH_2_-N), 46.2 (CH_2_-N), 36.0 (CH_2_-CO), 40.4 (CH_2_-N), 42.5 (CH_2_-N), 32.9 (CH_2_-N), 55.6 (CH_2_-N), 67.1 (CH_2_-O), 113.0 (Ar), 119.54 (Ar_ipso_), 127.4 (pyrrole), 135.7 (Ar), 157.7 (Ar_ipso_), 124.8 (C=C), 154.8 (C=C), 162.8 (C=O), 164.9 (C=O), 170.0 (C=O), 172.3 (C=O), 173.7 (C=O). MS MALDI TOF *m/z*: 3,764 (M^+^). Anal. Calcd. for C_180_H_322_N_64_O_24_. C 57.39%, H 8.62%, N 23.80%. Found: C 57.36; H 8.59; N 23.82%.

### 3.6. Synthesis of Dendrimers **6** and **7** with Fluorene in the Periphery

To a solution of dendrimers 3 and 5 (0.5 mmol) in methanol (15 mL), fluorene-2-carboxaldehyde (0.5 mmol) in methanol (15 mL) was added, the mixture was stirred in a nitrogen atmosphere at 45–50 °C for 3 day. The methanol was removed *in vacuo*. The residue was washed several times with methanol first and then with CH_2_Cl_2_ to obtain the desired products 6 and 7.

*Dendrimer* **6**. Purple solid, 85% yield. m.p: ˃300 °C. FTIR (pellet, KBr, cm^−1^): 3297, 2938, 1651, 1545, 1470, 1348, 1290, 1235, 1176, 1120, 797, 731, 587. UV-vis (CH_2_Cl_2_): λ_max_ 206, 426, 518, 559, 603, 647. ^1^H-NMR (CDCl_3_) δ_H_: −2.79 (br, 2H, NH), 1.90–1.96 (m, 16H, CH_2_-N), 3.30–3.54 (m, 16H, CH_2_-N), 4.06 (br, 16H, CH_2_-fluorene), 4.90 (s, 8H, CH_2_-O), 7.41 (br, 8H, Ar), 7.57–8.40 (br, 56H, fluorene, Ar-O), 8.08–8.11 (br, 8H, Ar), 8.85 (br, 8H, pyrrole). ^13^C-NMR (CDCl_3_) δ_C_: 38.8 (CH_2_-NH_2_), 40.4 (CH_2_-N), 41.6 (CH_2_-N), 42.2 (CH_2_-fluorene), 43.4 (CH_2_-N), 53.7 (CH_2_-N), 57.8 (CH_2_-N), 58.4 (CH_2_-O), 69.1 (CH_2_-O) 108.6 (Ar), 115.0 (Ar_ipso_), 137.3 (pyrrole), 164.6 (C=O), 171.9 (C=O), 175.2 (C=O), 175.6 (C=O). MS MALDI TOF *m/z*: 2,659 (M^+^). Anal. Calcd. for C_180_H_146_N_16_O_8_. C 81.24%, H 5.53%, N 8.42%. Found: C, 81.26; H, 5.55; N 8.40%.

*Dendrimer* **7**. Purple oil, 82% yield. (film, cm^−1^): 3257, 3074, 1644, 1547, 1433, 1368, 1293, 1239, 1178, 1043, 998, 642, 597. UV-vis (CH_2_Cl_2_): λ_max_ 209, 418, 517, 555, 593, 648. ^1^H-NMR (CDCl_3_) δ_H_: −2.86 (br, 2H, NH), 2.71 (br, 64H, CH_2_-N), 3.36 (br, 96H, CH_2_-N), 3.77 (br, 64H, CH_2_-N), 4.06 (br, 64H, CH_2_-fluorene), 4.80 (br, 8H CH_2_-O), 7.36 (br, 8H, Ar-O), 7.57–8.40 (br, 232H, fluorene, Ar-O), 8.79 (m, 8H, pyrrole), 9.39 (br, 32H, CH=N). ^13^C-NMR (CDCl_3_) δ_C_: 29.4 (CH_2_-NH_2_), 35.7 (CH_2_-N), 37.4 (CH_2_-N), 42.2 (CH_2_-fluorene), 45.1 (CH_2_-N), 48.7 (CH_2_-N), 50.3 (CH_2_-N), 56.4 (CH_2_-N), 60.4 (CH_2_-N), 67.3 (CH_2_-O), 112.8 (Ar), 119.1 (Ar_ipso_), 121.7 (Ar), 122.57 (Ar), 123,7 (Ar), 124.3 (Ar), 125.5 (Ar), 125.8 (Ar), 126.3 (Ar), 126.9 (Ar), 128.5 (pyrrole), 130.1 (Ar), 130.5 (Ar), 130.6 (Ar), 131.0 (Ar), 135.4 (Ar), 156.6 (CH=N), 168.6 (C=O), MS MALDI TOF *m/z*: 9405 (M^+^). Anal. Calcd. for C_628_H_578_N_64_O_24_. C 80.19%, H 6.19%, N 9.53%. Found: C 80.16; H 6.19; N 9.52%.

## 4. Conclusions

Two new classes of dendrimers bearing 8 and 32 fluorene donor groups have been synthesized. The first and second generation porphyrin-PAMAM-fluorene dendrimers were obtained in good yields. The UV-vis spectra showed that the individual properties of donor and acceptor moieties are preserved, indicating that the new dendrimers could be used as photosynthetic antennae. Also, for fluorescence spectroscopy, these dendrimers showed an efficient energy transfer.
